# A new method of arterial stiffness measurement in pseudoexfoliation syndrome: cardio-ankle vascular index

**DOI:** 10.5935/0004-2749.20220085

**Published:** 2025-02-11

**Authors:** Mahmut Atum, İbrahim Kocayiğit, Salih Sahinkuş, Şule Bahadır Coşkun, Erkan Çelik

**Affiliations:** 1 Department of Ophthalmology, Sakarya University Faculty of Medicine, Sakarya, Turkey; 2 Department of Cardiology, Sakarya University Faculty of Medicine, Sakarya, Turkey

**Keywords:** Cardio-ankle vascular index, Arterial stiffness, Exfoliation syndrome, Índice vascular coração-tornozelo, Rigidez vascular, Síndrome de exfoliação

## Abstract

**Purpose:**

To investigate whether pseudoexfoliation syndrome affects arterial stiffness by using cardio-ankle vascular index measurement.

**Methods:**

This cross-sectional case-control study included 55 patients with pseudoexfoliation syndrome and 106 ageand gender-matched healthy control subjects. All subjects underwent a complete ophthalmic exa mination of both eyes and cardio-ankle vascular index measu rements. Echocardiographic and body mass index measurements were performed in all patients, and the results were recorded. A binary regression model was used to determine the relationship between cardio-ankle vascular index and pseudoexfoliation.

**Results:**

There were no significant differences between the pseudoexfoliation and control groups in baseline clinical and demographic characteristics, echocardiographic measurements of left ventricular ejection fraction, and body mass index. The mean cardio-ankle vascular index value was significantly higher in the pseudoexfoliation group than in the controls (9.47 ± 1.23 vs. 8.33 ± 1.50, p<0.001). Intraocular pressure was significantly higher in the pseudoexfoliation group than in the controls (18.31 ± 1.78 vs. 15.24 ± 2.42 mm Hg, p<0.05). Although the logistic regression analysis showed that mean cardio-ankle vascular index and IOP values were positively associated with pseudoexfoliation syndrome (Odds ratios (OR) = 1.973, 95% CI, 1.051-3.706, p=0.035; OR=3.322, 95% CI = 2.000-5.520, p<0.001, respectively), the Pearson correlation analysis revealed a borderline significant positive correlation between age and mean cardio-ankle vascular index and a significant positive correlation between dyslipidemia and intraocular pressure and mean cardio-ankle vascular index (r=0.265, p=0.050; r=0.337, p=0.012; r=0.433, p=0.001, respectively).

**Conclusion:**

Our findings demonstrated that cardio-ankle vascular index values increased in patients with pseudoexfoliation syndrome.

## Abstract (pt)

**Objetivo:**

Investigar se a síndrome de pseudoesfoliação afeta a rigidez arterial, usando a medição do índice vascular cardíaco-tornozelo.

**Métodos:**

Este estudo transversal caso-controle incluiu 55 pacientes com síndrome de pseudoesfoliação e 106 controles saudáveis, pareados por idade e gênero. Todos os indivíduos foram submetidos a um exame oftalmológico completo de ambos os olhos e à medição do índice vascular cardíaco-tornozelo. Medidas ecocardiográficas e do índice de massa corporal também foram feitas em todos os pacientes, e os resultados foram registrados. Usou-se um modelo de regressão binária para avaliar uma possível relação entre o índice vascular cardíaco-tornozelo e a pseudoesfoliação.

**Resultados:**

Não houve diferença significativa entre os grupos com pseudoesfoliação e de controle em relação às características clínicas e demográficas basais, às medidas ecocardiográficas da fração de ejeção do ventrículo esquerdo e ao índice de massa corporal. Os valores médios do índice vascular cardíaco-tornozelo foram significativamente maiores no grupo com pseudoesfoliação do que no de controle (9,47 ± 1,23 contra 8,33 ± 1,50, p<0,001). Os valores da pressão intraocular no grupo com pseudoesfoliação excederam significativamente os do grupo de controle (18,31 ± 1,78 mmHg contra 15.24 ± 2.42 mmHg, p<0.05). A análise de regressão logística demonstrou uma associação positiva das médias do índice vascular cardíaco-tornozelo e da pressão intraocular com a síndrome de pseudoesfoliação (respectivamente, OR=1,973, IC 95%: 1,051-3,706, p=0,035 e OR=3,322, IC 95%: 2,000-5,520, p<0,001). Já a análise de correlação de Pearson revelou uma correlação positiva de significância limítrofe entre a idade e a média do índice vascular cardíaco-tornozelo, e uma correlação positiva significativa entre a dislipidemia, a pressão intraocular e a média do índice vascular cardíaco-tornozelo (respectivamente, r=0,265, p=0,050; r=0,337, p=0,012; e r=0,433, p=0,001).

**Conclusão:**

Nossos achados demonstraram que os valores do índice vascular cardíaco-tornozelo se encontram aumentados em pacientes com síndrome de pseudoesfoliação.

## INTRODUCTION

Pseudoexfoliation (PEX) syndrome is an age-dependent disorder characterized by an accumulation of abnormal fibrillary material in the extracellular matrix, usually associated with open-angle glaucoma^(1-3)^. PEX accumulation in the ocular tissues may be unilateral or bilateral in the anterior structures of the eye, such as the anterior capsule of the lens, zonules, and iris^(4)^. As shown in several studies, PEX material can accumulate in non-eye structures, such as the skin, lung, heart, liver, gallbladder, kidney, ear, optic nerve, and meninges^(5,6)^. PEX material also accumulates in the vessel walls, and there is an increased risk of coronary artery disease in people with PEX syndrome^(7,8)^.

Studies have shown that lysyl oxidase-like 1 (LOXL1) expression is irregular in PEX syndrome^(9)^. Whereas transient up-regulation of LOXL1 at the onset of the disease causes abnormal PEX accumulation, it has been shown that the decreased expression of LOXL1 in the later stages of the disease may adversely affect elastin metabolism. It has been proven that impairment of elastin metabolism may be associated with arterial stiffness^(10)^.

Arterial stiffness has been defined as the loss of elasticity of the arteries when subjected to vessel wall expansion. It is recognized as an independent marker of prognosis in persons with cardiovascular disease^(11,12)^. Arterial stiffness has been shown to increase the risk of cardiovascular conditions, dementia, and death in the elderly population^(13)^. Several anatomical and physiological parameters are used to evaluate arterial stiffness, including carotid intima média thickness and pulse wave velocity (PWV)^(14)^. Unlike other measures, the cardio-ankle vascular index (CAVI) allows physiological evaluation of aortic stiffness and is unaffected by the patient’s blood pressure^(15)^. CAVI measurement devices are widely used, especially in Japan, because of their portability, reproducibility, and ease of use. Studies have reported that CAVI has good reproducibility^(16)^.

The present study aimed to measure the arterial stiffness of patients with PEX syndrome using a CAVI device and compare the measurements with those in an ageand gender-matched healthy control group. Thus, we have investigated whether PEX syndrome affects arterial stiffness. Unlike previous studies, our study used the CAVI method, which is a noninvasive method of measuring arterial stiffness. To our knowledge, our study is the first to use this method in the PEX population.

## METHODS

### Ethical approval

Informed consent was obtained from the patients, and the approval of the local ethics committee (Sakarya University Faculty of Medicine Ethics Committee) was obtained before conducting the study. The study was conducted according to the principles of the Declaration of Helsinki.

This cross-sectional, case-control study was conducted at Sakarya University Training and Research Hospital, Ophthalmology, and Cardiology Clinics between January and December 2019. All subjects underwent a complete ophthalmic examination of both eyes, including best-corrected visual acuity (Snellen chart), slit-lamp examination of the anterior segment, intraocular pressure measurement (Goldmann applanation tonometry), and slit-lamp examination of the fundus with a 90D Volk lens. In addition, all patients underwent retinal nerve fiber layer analysis by optical coherence tomography (Cirrus HD OCT, Carl Zeiss Meditec, Dublin, CA, USA) and visual field analysis (Humphrey Field Analyzer program 30-2 test, Carl Zeiss Meditec, San Leandro, CA, USA). The diagnosis of PEX was based on the presence of gray-white-colored PEX material on the front capsule or the pupil edge of the lens of at least one eye by biomicroscopic examination. The control patients were patients admitted to the ophthalmology clinic for mild cataract (n=26) or presbyopia (n=80). Both eyes of the control patients were dilated, and no PEX material was detected.

Patients with a history of ocular trauma, PEX glaucoma, corneal diseases that precluded anterior segment imaging (neovascularization, dystrophy, leukoma, etc.), or previous ocular surgery, active ocular disease (uveitis, conjunctivitis, keratitis), or use of drugs that may reduce intraocular pressure were excluded from the study. Patients with cataracts at a level that would affect image acquisition and visual field testing were excluded from the study. Those with an accompanying disease that could affect CAVI measurements, such as peripheral artery disease, uncontrolled diabetes, renal failure, heart failure, and moderate or severe heart valve disease, were excluded from the study.

All measurements and echocardiographic examinations were performed by a single cardiologist (I.K). Basic parameters, such as left ventricular diastolic and systolic diameters, left ventricular wall thickness, and ejection fraction, were obtained following the current recommendations^(17)^. Left ventricular ejection fraction was calculated by a modified Simpson’s method. Complete two-dimensional, motion mode (m-mode), color, and spectral Doppler echocardiography were performed from standard imaging planes with a Phillips EPIQ CVx ultrasound machine with the use of a 1.5-4.6 MHz transducer (Philips Healthcare, Andover, MA, USA).

### Cardio-ankle vascular index measurement

Noninvasive measurement of CAVI was performed with a VaSera VS-1000 device (Fukuda Denshi Co. Ltd, Tokyo, Japan). CAVI measurements were performed with the patient in the supine position. After the subject had rested for 10 min, blood pressure cuffs were wrapped around the four extremities to measure blood pressure and detect upper arm and ankle pulse waves. Electrocardiographic electrodes were placed on both wrists, and a microphone was placed in the sternum (second intercostal space) for phonocardiography^(18)^. The CAVI measurement took approximately 10 min for each patient, and the results were recorded. CAVI was calculated by the device using its internal algorithm^(18)^.

The CAVI measurement gives us information about the stiffness of large arteries^(19,20)^. The VaSera device calculates the CAVI values automatically by the formula CAVI = a[(2ρ/∆P) × In(Ps/Pd)PWV2] + b, where Pd is diastolic blood pressure, Ps is systolic blood pressure, ∆P is Ps - Pd, ρ is blood density, in is natural logarithm (log_e_), and a and b are constants. The ankle-brachial index (ABI) was calculated by dividing systolic blood pressure at the ankle by that of the brachial artery. The ABI is used in clinical practice to assess the patency of the lower limb arterial system and to screen for the presence of obstructive peripheral artery disease. CAVI is disregarded if the ABI is below 0.9^(19,21)^. Subjects with ABI below 0.90 were excluded from the study, because patients with severe peripheral arterial occlusive diseases may yield false results in CAVI measurements.

### Statistical analysis

The data were analyzed by SPSS 20.0 (Statistical Package for Social Sciences) statistical software (SPSS, Chicago, IL, USA). Continuous variables were expressed as means ± SD, and categorical variables were expressed as percentages. The Kolmogorov-Smirnov test was used to check the distribution of continuous variables, and the distribution was found to be normal. Categorical variables were compared between the two groups by the chi-squared test. Student’s *t-*test was used to compare continuous variables. A binary regression model was applied to determine a possible relationship between CAVI and PEX. Odds ratios (ORs) were estimated with 95% confidence intervals (95% CI). Pearson correlation analysis was used to analyze the relationship between CAVI/ABI and patient characteristics, and p<0.05 was considered to indicate statistical significance.

## RESULTS

The study included 55 patients diagnosed with PEX syndrome and 106 control subjects. Table 1 shows the demographic and clinical characteristics of the two groups. There were no significant differences between the PEX and control groups in clinical and demographic characteristics, echocardiographic measurements of the left ventricular ejection fraction, and BMI. The mean MD and PSD were -0.9 ± 1.1 dB and 1.4 ± 0.4 dB in the control group and -1.1 ± 1.4 dB and 1.5 ± 1.1 dB in the PEX group, respectively (p>0.05, p>0.05, respectively).

**Table 1:** Comparison of demographic and clinical data of the PEX group and the control group

Parameter	PEX group (n=55)	Control group (n=106)	P value
Male sex (n, %)	31 (56.4)	49 (46.2)	0.22
Age (mean±SD, years)	68.6 ± 8.4	67.3 ± 7.4	0.31
BMI (mean±SD, kg/m^2^)	27.9 ± 3.8	29.1 ± 4.9	0.15
DM (n, %)	11 (20)	33 (31.1)	0.13
HT (n, %)	32 (58,2)	74 (69,8)	0.14
CAD (n, %)	10 (18,2)	15 (14,2)	0.50
Current smoking	14 (25,5)	21 (19,2)	0.41
Systolic BP (mean±SD, mmHg)	138.9 ± 10.1	137.9 ± 14.2	0.68
Diastolic BP (mean±SD, mmHg)	81.9 ± 6.4	82.4 ± 10.7	0.80
Dyslipidemia (n, %)	12 (21,8)	15 (14,2)	0.37
LVEF (mean±SD, %)	64.1 ± 5.9	62.7 ± 4.1	0.13

BMI= body mass index; BP= blood pressure; CAD= coronary artery disease; DM= diabetes mellitus; HT= hypertension; LVEF= left ventricular ejection fraction; PEX= pseudoexfoliation.

The mean CAVI values were significantly higher in the PEX group than in the control group (9.47 ± 1.23 vs. 8.33 ± 1.50, p<0.001). Both right and left CAVI values were significantly higher in the PEX group than in the control group (9.50 ± 1.30 vs. 8.32 ± 1.57, p<0.001; 9.45 ± 1.31 vs. 8.34 ± 1.48, p<0.001, respectively) (Table 2). There were no significant differences between the PEX and control groups in mean, right, and left ABI values (1.08 ± 0.13 vs. 1.05 ± 0.11, p=0.260; 1.08 ± 0.14 vs. 1.06 ± 0.11, p=0.285; 1.07 ± 0.13 vs. 1.05 ± 0.12, p=0.282, respectively). The IOP values were significantly higher in the PEX group than in the control group (18.31 ± 1.78 mmHg [range, 14-21] vs. 15.24 ± 2.42 mmHg [range, 11-20], p<0.001].

**Table 2:** Comparison of CAVI, ABI, and IOP in the PEX group and the control group

Variable	PEX syndrome (n=55)		Control (n=106)		P value^*^
CAVI (right)	9.50 ± 1.30		8.32 ± 1.57		<0.001
CAVI (left)	9.45 ± 1.31		8.34 ± 1.48		<0.001
CAVI (mean)	9.47 ± 1.23		8.33 ± 1.50		<0.001
ABI (right)	1.08 ± 0.13		1.06 ± 0.11		0.285
ABI (left)	1.07 ± 0.13		1.05 ± 0.12		0.28
ABI (mean)	1.08 ± 0.13		1.05 ± 0.11		0.26
IOP	18.31 ± 1.78		15.24 ± 2.42		<0.001

ABI= ankle-brachial index; CAVI= cardio-ankl pressure; PEX= pseudoexfoliation.

* Student’s *t*-test.

Binary logistic regression analysis was performed to find independent factors associated with PEX syndrome. Age, CAVI (mean), ABI (mean), IOP, BMI, sex, diabetes mellitus, hypertension, dyslipidemia, smoking, and coronary artery disease were included in the equation. Mean CAVI values and IOP measurements were positively associated with PEX syndrome (OR=1.973, 95% CI, 1.051-3.706, p=0.035; OR=3.322, 95% CI = 2.0005.520, p<0.001, respectively) (Table 3).

**Table 3:** Binary logistic regression analysis

Independent variable	OR	95% Cl for OR (lower)	95% CI for OR (upper)	P value
Age	0.99	0.90	1.09	0.86
CAVI (mean)	1.97	1.05	3.71	**0.03**
ABI (mean)	0.01	0.00	1.93	0.09
1OP	3.32	2.00	5.52	**<0.05**
BMl	1.12	0.93	1.34	0.24
Sex	0.81	0.17	3.76	0.78
DM	1.62	0.24	10.84	0.62
HT	3.35	0.47	23.65	0.22
Dyslipidemia	0.21	0.02	1.76	0.15
Smoking	0.40	0.06	2.73	0.34
CAD	1.32	0.13	12.88	0.81

The dependent variable is PEX syndrome.

ABI= ankle-brachial index; BMI= body mass index; CAD= coronary artery disease; CAVI= cardio-ankle vascular index; CI= confidence interval; DM= diabetes mellitus; HT= hypertension; IOP= intraocular pressure; OR= odds ratio; PEX= pseudoexfoliation.

Pearson correlation analysis was performed between patient characteristics and mean CAVI and ABI values in the PEX syndrome and control groups. There was a borderline significant positive correlation between age and mean CAVI, and there was a significant positive correlation between dyslipidemia and IOP and mean CAVI (r=0.265, p=0.050; r=0.337, p=0.012; r=0.433, p=0.001, respectively) (Table 4). In the control group, there were significant positive correlations among age, coronary artery disease, smoking, and mean CAVI (r=0.302, p=0.02; r=0.210, p=0.031; r=0.043, p=0.015; r=0.236, p=0.015, respectively).

**Table 4:** Correlation between mean CAVI, mean ABI, and PEX syndrome (Pearson correlation test)

Parameter	CAVI (mean)		ABI (mean)	
	r	P value	r	P value
Age	0.265	**0.05**	-0.13	0.345
Sex	-0.14	0.31	-0.09	0.50
DM	0.15	0.26	0.12	0.375
HT	0.22	0.10	-0.17	0.21
CAD	0.19	0.16	0.11	0.42
Dyslipidemia	0.34	**0.01**	0.002	0.99
BMI	-0.17	0.22	0.05	0.72
Smoking	-0.085	0.54	-0.105	0.45

ABI= ankle-brachial index; BMI= body mass index; CAD= coronary artery disease; CAVI= cardio-ankle vascular index; DM= diabetes mellitus; HT= hypertension; PEX= pseudoexfoliation.

## DISCUSSION

In our study, we hypothesized that PEX materials that accumulate in the vessel walls may also accumulate in major arteries such as the aorta, and therefore, aortic stiffness may be greater in patients diagnosed with PEX syndrome than in the control group with similar characteristics.

There are several possible mechanisms to explain the relationship of arterial stiffness with PEX syndrome. Histopathological examination of the postmortem aortic wall of individuals with PEX syndrome showed accumulation of PEX material in adventitial and subendothelial connective tissue, as well as pronounced fibrosis and tunica intima elastosis. It is believed that fibroblast cells in connective tissue may be responsible for the production of PEX material. This causes damage to the vessel wall, resulting in hardening of the vessels^(22)^.

Many studies have investigated the relationship between PEX syndrome and various systemic diseases. Mitchell et al. surveyed 3,546 individuals in a large-scale community-based study called The Blue Mountains Eye Study. They found a significant increase in vascular risk in those with PEX syndrome and a significant relationship with a history of angina or hypertension or a combined history of angina, acute myocardial infarction, and stroke^(8)^. In another study conducted by Citirik et al., according to the results of coronary angiography, patients with and without CAD were compared in terms of the presence of PEX. In the CAD group, PEX syndrome was found to be significantly more common^(23)^. Another large-scale study of 6,046 patients with PEX syndrome found that ischemic heart disease, cardiomyopathy, and aortic aneurysms were significantly more common in patients with PEX^(24)^. All these studies suggest that the accumulation of PEX material in the vessel walls causes arterial stiffness by disrupting the elasticity of the large vessels.

Some studies have investigated whether arterial stiffness increases in patients with PEX syndrome and glaucoma. Visontai et al. compared baroreflex sensitivity among 30 patients with the PEX syndrome and glaucoma and the control group, and found that common carotid artery stiffness increased significantly in the PEX group^(25)^. Türkyılmaz et al. compared 25 patients with PEX glaucoma with 25 control subjects and looked at the carotid-femoral PWV values of the patients. The results showed that carotid-femoral PWV values increased significantly in the PEX glaucoma group^(26)^. In our study, CAVI was used as a measurement method, and similar results were obtained to those of Türkyılmaz et al. All these results suggest that there is a relationship between PEX syndrome and vascular damage.

Chiba et al. compared brachial-ankle PWV (baPWV) values in three groups of patients with glaucoma (normal-tension glaucoma, primary open-angle glaucoma, and ocular hypertension) with baPWV values in a control group and found no significant differences^(27)^. To investigate systemic artery stiffness in patients with glaucoma and control patients with diabetes mellitus, Shim et al. measured baPWV and found that baPWV was significantly greater in the group with glaucoma^(28)^. The difference between the results of the two studies may be due to the difference in patient groups.

Several methods can be used to measure arterial stiffness, one of which is the widely used PWV method^(12)^. Kabutoya et al. measured CAVI in 4,545 patients with at least one cardiovascular risk factor and baPWV in 1,737 of these patients. There was a significant positive correlation between CAVI and baPWV (r=0.50, p<0.001)^(29)^. However, PWV is well known to depend on blood pressure at the time of measurement. Furthermore, CAVI has some advantages over other methods. It does not require a probe in the neck or groin and is mostly operator-independent, it reflects the stiffness of the entire aorta and is unaffected by blood pressure during measurement, and its reproducibility is good^(30)^. In this respect, we believe that CAVI measurement may be a more reliable method for revealing arterial stiffness.There are some limitations to our study. There was a small number of subjects in the patient group. The duration of hypertension and cardiovascular diseases and the use of antihypertensive and antidyslipidemia medications can also affect arterial stiffness. However, no data were collected on the use of medications or disease duration. The lack of patients with PEX glaucoma was also a limitation.

In summary, the results showed that patients with PEX syndrome had higher CAVI than controls. We suggest that there may be a link between PEX syndrome and arterial stiffness, since high CAVI is associated with arterial stiffness.
